# Effects of Organic Acids on Phenolic Compounds and Antioxidant Activity During the Processing of Potatoes with Colored Flesh

**DOI:** 10.3390/molecules31172948

**Published:** 2026-08-22

**Authors:** Anna Pęksa, Agnieszka Tajner-Czopek, Kamila Marciniak, Anna Sokół-Łętowska, Agnieszka Nemś, Alicja Zofia Kucharska

**Affiliations:** 1 Department of Food Storage and Technology, Faculty of Biotechnology and Food Science, Wrocław University of Environmental and Life Sciences, Chełmońskiego St. 37, 51-630 Wrocław, Poland; anna.peksa@upwr.edu.pl (A.P.); kamila.marciniak29081999@gmail.com (K.M.); agnieszka.nems@upwr.edu.pl (A.N.); 2 Department of Fruit, Vegetable and Plant Nutraceutical Technology, Faculty of Biotechnology and Food Science, Wrocław University of Environmental and Life Sciences, Chełmońskiego St. 37, 51-630 Wrocław, Poland; anna.sokol-letowska@upwr.edu.pl (A.S.-Ł.); alicja.kucharska@upwr.edu.pl (A.Z.K.)

**Keywords:** anthocyanins, phenolic acids, potatoes with colored flesh, dehydrated potato grits, organic acids, antioxidant activity, ABTS, DPPH, FRAP

## Abstract

Potatoes with colored flesh are increasingly processed in the food industry. However, establishing appropriate production parameters is challenging because anthocyanins and other polyphenols present in potatoes undergo structural transformations during processing. Therefore, this study investigated the effects of five organic acids applied during the production of dehydrated potato grits on the content, profile, and stability of phenolic compounds in the final products manufactured from tubers of the red-fleshed potato cultivar Lily Rose (LR) and the purple-fleshed cultivar Double Fun (DF). The effects were assessed by analyzing the profiles, antioxidant activity, and content of the phenolic compounds in the final products. Phenolic compounds were analyzed by high-performance liquid chromatography (HPLC), while antioxidant activity was evaluated using the FRAP, DPPH, and ABTS assays. Only malic acid improved anthocyanin retention in LR potato products, while tartaric and malic acids had the greatest effect on the content of the analyzed compounds in DF potato products. Citric acid showed only a moderate stabilizing effect on anthocyanin content. Grits produced with malic and tartaric acids contained 20–67% more phenolic acids than the control and showed higher antioxidant activity. Lactic acid had an adverse effect, particularly in LR dehydrated potato grits. Depending on the potato cultivar, the effectiveness of organic acids in stabilizing anthocyanins varied. Therefore, the processing conditions for colored potatoes should be optimized for the specific cultivar.

## 1. Introduction

Potatoes with purple or red flesh, which contain anthocyanins—compounds with proven antioxidant properties and beneficial effects on human health—are attracting increasing interest from scientists, food producers and consumers [1,2,3]. Compared to cultivars with traditional cream or white flesh, colored potatoes are characterized by phenolic compound contents that are several times higher, ranging from 461 to 946 mg GAE 100 g^−1^ DW [2,4,5,6]. The amounts of these compounds determined, for example, in dried vegetables reach 526 mg GAE/100 g in beetroot, 109 mg GAE/100 g in red cabbage, and 597 mg GAE/100 g in red pepper, according to Kosewski et al. [7]. Potato cultivars with colored flesh differ significantly in their anthocyanin content and profile. Several authors [8,9] confirm that red-fleshed cultivars contain fewer anthocyanins than purple-fleshed cultivars. Among red-fleshed cultivars, these compounds are represented primarily by pelargonidin [5,10,11,12], including the particularly prevalent pelargonidin-3-rutinoside-5-glucoside, acylated *p*-coumaric acid, which is the main anthocyanin (ca. 70%). In purple-fleshed potato cultivars, petunidin and malvidin derivatives predominate [2,3]. The susceptibility of anthocyanins to factors such as pH, heating, enzymes, access to oxygen, metal ions, or white light is known [8,13,14,15,16]. Among the numerous phenolic compounds found in colorful potatoes, phenolic acids also play an important health-promoting role, demonstrating, among other things, significant antioxidant and antiradical activity. This is confirmed by research results from numerous authors [2,17,18,19,20]. The dominant phenolic acid in potatoes, including those with colored flesh, is chlorogenic acid. Its proportion in the tuber skin can reach up to 90% [5,9,18,19,21]. It occurs in three isomeric forms: chlorogenic acid (5-*O*-caffeoylquinic acid; 5-CQA), neochlorogenic acid (3-*O*-caffeoylquinic acid; 3-CQA) and cryptochlorogenic acid (4-*O*-caffeoylquinic acid; 4-CQA) [18,20]. Other phenolic acids found in potatoes include *p*-coumaric, ferulic, gallic, and caffeic acids [5,21,22,23]. Phenolic acids in potatoes are unstable during prolonged drying of potato slices or during specific frying (deep-frying while stirring). Under such conditions, they undergo oxidation, and the activity of these compounds in the final products remains minimal [20,24]. Chlorogenic acid content has been found to decrease during processing, but to a greater extent in white potatoes than in pigmented potatoes [25]. When processing potatoes containing anthocyanins, the susceptibility of these compounds to degradation under processing conditions should be considered, and appropriate modifications to the processing technology should be implemented [2,13,26,27,28]. Dehydrated potatoes are an important product in the food industry. They are a key ingredient in various snacks and dishes [2]. One of the production steps in the dehydrated potato production process is blanching the potato slices in hot water, often with the addition of antioxidants [29]. The elevated temperature of this process inhibits the activity of oxidative enzymes, primarily polyphenol oxidase, while the antioxidants used, such as SO_2_, prevent darkening of the flesh of raw tubers and the dried products obtained from them [30]. However, the use of SO_2_ in the production of dehydrated potatoes containing anthocyanins is limited because this compound causes unfavorable changes in their final color [13,29,30]. The use of organic acids in the production of dehydrated potato grits can partially prevent the degradation of anthocyanins and can have a positive effect on the color of these products. The influence of tartaric and citric acids was demonstrated by Nemś et al. [13] and Pęksa et al. [29] in studies on the use of colored potatoes in the production of snacks. Sampaio et al. [28] studied the color and anthocyanin stability in potato juices from seven cultivars with colored flesh that had been pasteurized and treated with citric acid.

Research by Lv et al. [16] indicated that significant differences in the color of products containing anthocyanins induced by organic acids are related to changes in the structure of these compounds. Processing conditions for potatoes with colored flesh, including the use of elevated temperature and variable pH, may, among other things, lead to the degradation of anthocyanins and the loss of their specific properties. Studies by various authors [14,15,31,32] indicate that the stabilization of anthocyanins under the processing conditions of plant materials is influenced by co-pigmentation reactions involving anthocyanins and other compounds, including flavanols, metal ions, polysaccharides, and organic acids, including phenolic acids.

The stabilization of anthocyanins with the participation of various organic acids in technological processes of food production is still relatively poorly researched, probably due to the influence of many different factors, often simultaneously. Depending on the degree of glycosylation and acylation, as well as the type of phenolic acid and/or organic acid involved in acylation, anthocyanin stability under processing conditions may vary [26,33].

There is limited information on the effect of thermal processing of colored potatoes on their color and on the changes occurring in their anthocyanins. To the authors’ knowledge, the stabilisation of cyanidin-3-glucoside anthocyanin using organic acids during fermentation was investigated by Wu et al. [34]. However, there is a lack of research on the stabilization of these compounds during potato processing using various organic acids.

Therefore, this study investigated the effects of five organic acids applied during the production of dehydrated potato grits on the content, profile, and stability of phenolic compounds, including anthocyanins and phenolic acids, in the final products obtained from potatoes of two cultivars with colored flesh, and evaluated the antioxidant activity of the resulting products. It was hypothesized that individual organic acids would differ in their ability to stabilize anthocyanins and other phenolic compounds during processing, and that these effects would depend on the potato cultivar, resulting in cultivar-specific differences in anthocyanin and phenolic acid retention and antioxidant activity.

## 2. Results and Discussion

### 2.1. Characteristics of the Raw Material

The tested potatoes differed slightly in terms of starch, total protein, and reducing sugar content (Table 1).

Due to their low reducing sugar content and medium starch content, tuber samples from both cultivars were the preferred raw material for producing French fries and dried products. From raw materials with such characteristics, it is possible to obtain potato products with the correct color and consistency [20,35]. In the processing of potatoes with a low content of reducing sugars (not higher than 0.15%), Maillard darkening reactions are significantly limited and have a lesser effect on the color of products from potato cultivars with red or purple flesh [36]. Larger differences were noted in terms of total polyphenol and flavonoid contents. Purple DF potatoes contained more total polyphenols (average approximately 438 mg GAE·100 g^−1^ DW), whereas red LR potatoes contained more flavonoids (average approximately 303 mg CE·100 g^−1^ DW).

Other authors [4,9,20,37,38] studying colored potatoes found the presence of phenolic compounds in their composition at levels ranging from 461 to 946 mg GAE·100 g^−1^ DW. They also confirmed that red- and purple-fleshed potatoes may contain up to twice as many flavonoids as white-fleshed cultivars. Research in this area confirms that some red-fleshed potato cultivars, such as Highland Burgundy Red, may contain more flavonoids than purple-fleshed cultivars. They attribute this to the influence of both color and cultivar on the content of these compounds. Regardless of flesh color, differences in the content of flavonoids such as catechin, kaempferol, quercetin, and myricetin may occur [38].

Significant differences were found in the DPPH antiradical activity and FRAP reducing power of the tested potatoes. DF cultivar potatoes were characterized by higher DPPH activity (25.55 µmol Trolox·g^−1^ DW) compared to LR cultivar tubers (22.55 µmol Trolox·g^−1^ DW). FRAP values were approximately 60% higher in DF cultivar potatoes. The studies by Cebulak et al. [39] showed that the antioxidant activity of red- and purple-fleshed potato cultivars varied depending on the color of the tuber flesh. These authors demonstrated the highest FRAP-reducing and DPPH-scavenging power of purple-fleshed Vitelotte potatoes. FRAP values were on average 114% higher than those of the yellow-fleshed Lord cultivar, and DPPH values were on average 6.4 times higher. Various authors [9,17,18,20,40] have confirmed the significant relationship between antioxidant activity and polyphenol content in potatoes. In the tested potatoes of the red-fleshed LR and purple-fleshed DF cultivars, various anthocyanins were identified, mainly acylated, and significant differences in the amounts of these compounds were demonstrated, depending on the potato cultivar (Table 2).

Based on the preliminary identification of individual anthocyanins, it was established that they are mainly acylated with coumaric acid. Brown et al. [41] found that more than 98% of the total anthocyanin content (TAC) in colored potatoes is acylated glycosides, which differ in acylation pattern by acid type. These are most often caffeic or coumaric acids but are also often replaced by ferulic acid [27,42].

The tested LR cultivar tubers with red flesh contained approximately fourfold lower anthocyanin levels than those of the DF tubers with purple flesh. In LR tubers, pelargonidin glycosides dominated, especially Pelargonidin-3-*p*-coumaroylrutinoside-5-glucoside (46.2 mg Cyanidin-3-*O*-glucoside·100 g^−1^ DW), whereas petunidin derivatives were the predominant anthocyanins in DF tubers. Of these, Petunidin-3-*p*-coumaroylrutinoside-5-glucoside was determined to be the most abundant (188.7 mg Cyanidin-3-*O*-glucoside·100 g^−1^ DW). Additionally, according to other authors [2,10,28], pelargonidin and peonidin glycosides predominate in red-fleshed potatoes. In their studies, these anthocyanins were present in amounts of 200–2000 mg·kg^−1^ FW and 20–200 mg·kg^−1^ FW, respectively. Brown et al. [41] found similar but also three times higher amounts of anthocyanins (69–350 mg·kg^−1^ FW) in tubers of red-fleshed potato cultivars than those determined in the LR potatoes we tested. Studies conducted by various authors [39,41] show that the amounts of anthocyanins determined in potato cultivars with colored flesh are not strictly related to the flesh color but rather to the cultivar. Purple-fleshed potato cultivars studied by Brown et al. [41] contained from 55 to 171 mg·kg^−1^ FW of anthocyanins, i.e., almost three times less than the purple-fleshed DF cultivar studied by us, while the purple-fleshed potatoes studied by Cebulak et al. [39] contained similar amounts of these compounds to our potatoes. These were mainly petunidin derivatives with an average content of 23.15 mg·100 g^−1^ FW, with the highest concentrations detected in the Vitelotte cultivar (51.27 mg·100 g^−1^ FW).

Four phenolic acids were initially identified in the tested raw material, including three isomers of chlorogenic acid (chlorogenic acid, neochlorogenic acid, cryptochlorogenic acid) and coumaric acid (Table 2). Numerous studies conducted by various authors [2,17,20,30,43] indicate that phenolic acids constitute the largest group of phenolic compounds in potatoes, accounting for approximately 90% of those in the skin. LR cultivar potatoes contained on average 26% more phenolic acids compared to DF cultivar tubers. Chlorogenic acid was found in the highest amounts, regardless of the potato cultivar (in the range of 146.9–179.7 mg 100 g^−1^ DW). A higher proportion of this acid (78%) in total phenolic acids was found in the DF cultivar. However, these potatoes had significantly lower amounts of other phenolic acids, particularly coumaric acid (2.14 mg·100 g^−1^ DW), whose proportion in total phenolic acids (1.1%) was 5 times lower than in LR cultivar tubers (5.4%). Additionally, in the five potato cultivars with colored flesh studied by Cebulak et al. [39], chlorogenic acid was dominant, constituting on average 96% of the determined phenolic acids. The potatoes examined by these authors contained on average about twice as many phenolic acids (over 89.19 mg·100 g^−1^ FW) as the potatoes of the LR and DF cultivars examined by us, while the content of coumaric acid (on average 0.64 mg·100 g^−1^ FW) was similar to that found in the DF tubers.

### 2.2. Effects of Organic Acids and Potato Cultivars on Polyphenol Content and Antioxidant Activity of Dehydrated Potato Grits

Dehydrated potato grits obtained from two potato cultivars, red- and purple-fleshed, differed in total polyphenol and flavonoid contents, antiradical activity (DPPH and ABTS) and antioxidant power—FRAP (Table 3).

**Figure 1:**
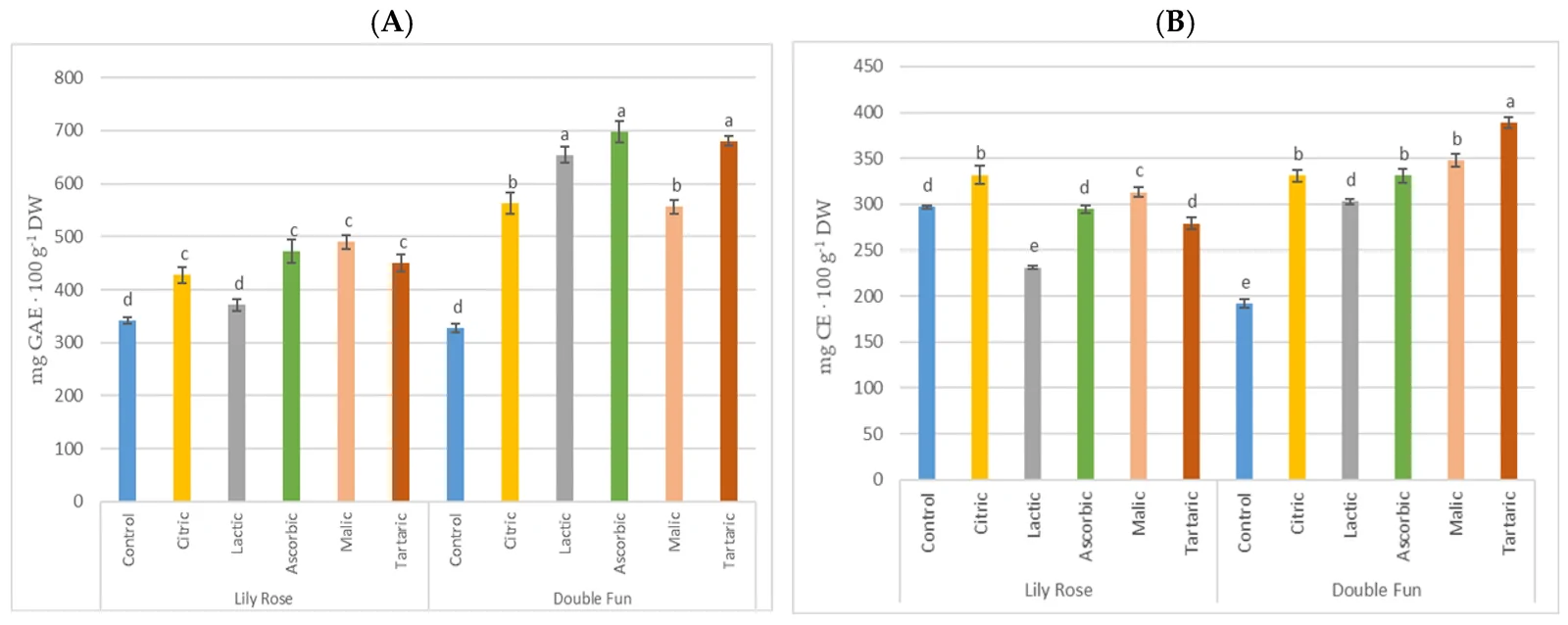
The effect of the type of organic acid on total polyphenol (**A**) and flavonoid (**B**) content of experimental grits obtained from potatoes of two cultivars; ±SD—standard deviation; *n* = 6; different lowercase letters (a–e) indicate significant differences (*p* < 0.05).

The research results indicate a beneficial effect of organic acids applied after blanching potato slices in hot water on the polyphenol content in the dehydrated products obtained from them. Regardless of the potato cultivar, grits obtained with organic acids, especially ascorbic, tartaric, and malic acids, contained more total polyphenols than the control sample (Table 3). In the case of dehydrated potato products from DF tubers, these differences reached, on average, 41–52%, while grits obtained with the aforementioned three acids from LR potatoes contained, on average, 20–30% more polyphenols than the control sample (Figure 1A).

Regardless of the type of acid used, DF dehydrated potato grits contained more flavonoids than the control sample. This was not observed in grits made from LR tubers. Only the use of citric and malic acids contributed to a higher content of flavonoids in these final products (Figure 1B). Studies have also shown the adverse effect of using lactic acid in the production of colored grits, especially from LR tubers. The results of the conducted research indicate that the way acids interact with phenolic compounds found in colored potatoes is strongly dependent on the phenolic matrix of a given cultivar. The results clearly indicate that the response of the phenolic system to the addition of organic acids is not universal. Cao et al. [44] also report that the stability of polyphenols may result from their structural changes, which occur during various chemical and biochemical reactions. These are often related to environmental conditions and external factors, such as light, pH, and temperature, as well as interactions with other components found in the raw material [45].

**Figure 2:**
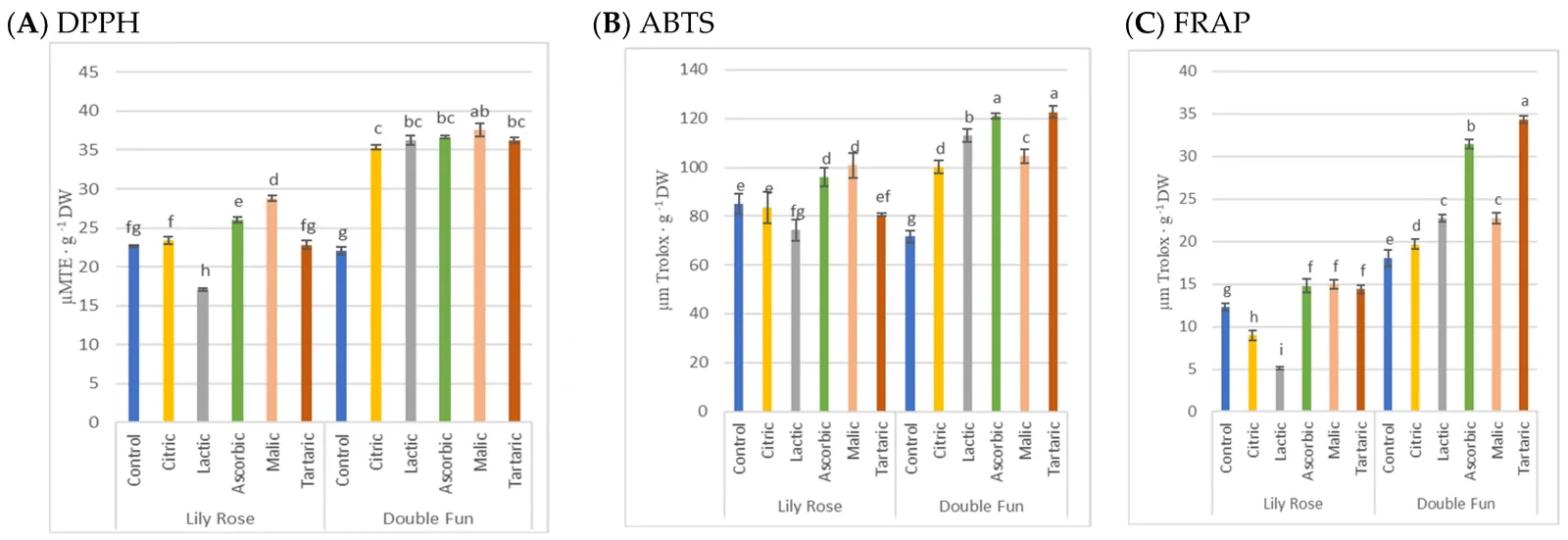
The effect of the type of organic acid on antioxidant and antiradical activity (**A**–**C**) of experimental grits obtained from potatoes of two cultivars; (±SD)—standard deviation; (*n* = 6); different lowercase letters (a–i) indicate significant differences (*p* < 0.05).

The antiradical activity determined by the DPPH and ABTS assays and the antioxidant capacity measured by the FRAP assay (Table 3) depended mostly on the potato cultivar, the type of organic acid used, and the interaction of these two factors. Other authors [2,3,9,46,47] found that purple- and red-fleshed potatoes differed in terms of antioxidant activity, depending on both the variety and the flesh color, and that these differences could be related primarily to the content and profile of anthocyanins. Compared to the control sample, the highest antiradical activity, expressed by both ABTS and DPPH, was observed in dried potato grits obtained with ascorbic, tartaric, and especially malic acids. However, the highest antioxidant activity (FRAP) was observed in products obtained with malic and tartaric acids (Table 3, Figure 2A–C). This was likely due to the fact that these acids enhanced the activity of anthocyanins [33].

Ascorbic acid significantly influenced the antiradical (DPPH) and antioxidant (FRAP) properties of the obtained dehydrated potato grits, which were higher than those of the control sample. This effect may be attributable to the additional antioxidant activity provided by ascorbic acid. Furthermore, the interaction between potato cultivar and acid type suggests that the anthocyanin acylation profile influences the system’s response to acid addition [16,29].

A smaller effect of the acids used on the antioxidant activity of LR potato products was observed, while a greater variability of antioxidant activity, depending on the type of acid, was noted in DF potato products. The DPPH and ABTS activities of DF dehydrated potato grits produced with organic acids were 38–41% and 28–42% higher, respectively, than the control sample. A beneficial effect on the DPPH and ABTS antiradical activity of LR potato products was observed after the application of ascorbic and malic acids. Compared to the control sample, the antiradical and antioxidant activities of dehydrated potato grits obtained using these two acids were higher by 11% and 16%, respectively (Figure 2A–C).

The observed differences in the antioxidant activity of dehydrated potato grits, depending on the potato cultivar, could have resulted from differences in the oxidative resistance of the dominant anthocyanins [38,40], i.e., the lower oxidative resistance of the anthocyanin Pelargonidin-3-*p*-coumaroylrutioside-5-glucoside in the LR cultivar potatoes and the higher oxidative resistance of the anthocyanin Petunidin-3-*p*-coumaroylrutinoside-5-glucoside in the DF cultivar tubers.

The FRAP antioxidant potential of experimental grits obtained with each of the five organic acids from DF tubers was significantly higher than that of the control sample (Figure 2C). The antioxidant properties of these finished product samples, expressed using FRAP, were on average 8–47% higher than those of the control sample. With respect to LR cultivar potato products, an increase in FRAP values was noted in samples prepared using ascorbic, malic, and tartaric acids (in the range of 15–18%). When citric and lactic acids were used, lower activity of the produced experimental grits was observed compared to the control sample.

### 2.3. Effect of Organic Acid Type and Potato Cultivar Used on the Content and Anthocyanin Profiles of Colored Dehydrated Potato Grits

Similar to the analyzed raw material, dehydrated potato grits from Double Fun tubers contained up to 80% more anthocyanins than grits from Lily Rose cultivar potatoes. In dehydrated grits from LR tubers, pelargonidin glycosides dominated, while in grits from purple DF tubers, petunidin and malvidin derivatives dominated. Lactic acid has been found to have an unfavorable effect on anthocyanin content, particularly in LR dehydrated potatoes, which may be attributed, at least in part, to its lower acidification capacity (pKa ~ 3.86) compared to other acids tested. In the case of other acids, an anthocyanin-stabilizing effect was noted. The positive effect of most of the acids used on anthocyanin structure stabilization may be related to their effects on lowering potato pH (citric acid), regulating/stabilizing pH (citric acid, tartaric acid), and antioxidant activity (ascorbic and citric acids) [10]. This is indicated, for example, by the studies of Fan et al. [48], who found that anthocyanins contained in fermented, purple-fleshed sweet potatoes are more stable in a strongly acidic environment (pH range 2.0–4.0) than in a slightly acidic environment (pH 5.0–6.0). Furthermore, Wu et al. [34] demonstrated that, at a constant pH, the number of carboxyl groups in an organic acid may also influence the color intensity of cyanidin-3-glucoside, indicating that structural characteristics of organic acids, in addition to their acidification capacity, can affect anthocyanin stability and color expression.

The effect of organic acid type on the anthocyanin profile and content in experimental grits was most pronounced in samples obtained from tubers of the purple-fleshed DF cultivar (Table 4).

These dehydrated potato grits contained two to three times more total anthocyanins than the control sample. This indicated that the anthocyanins found in DF potatoes, primarily Petunidin-3-*p*-coumaroylrutinoside-5-glucoside, were more susceptible to the structure-stabilizing effects of organic acids than the anthocyanins found in LR tubers, primarily Pelargonidin-3-*p*-coumaroylrutinoside-5-glucoside. In the experiment, malic and tartaric acids had the greatest stabilizing effect on anthocyanins and their activity in the dehydrated potatoes obtained from LR potatoes, whereas tartaric acid predominantly had the greatest stabilizing effect in the grits made from DF potatoes, where acylated petunidin dominated. This could be due to, among other things, the formation of multipoint hydrogen bonds or the “stiffening” of acylated residues (*p*-coumaroyl, feruloyl), which may contribute to enhanced anthocyanin stability. Dehydrated potato grits obtained from LR cultivar potatoes contained the highest levels of anthocyanins after the application of malic acid, which was probably due to its greatest stabilizing effect on pelargonidin glycosides present in the tubers of this cultivar (Table 4).

Tartaric acid used in the production of dehydrated potato grits of the DF cultivar showed a strong stabilizing effect on acylated anthocyanins, derivatives of petunidin, dominant in potatoes of this cultivar. The structure of anthocyanins directly influences the intermolecular interactions in the copigmentation process. Acylated and non-acylated anthocyanins are characterized by different chemical reactivity [31,33]. Lactic acid showed a variable and generally weaker effect on anthocyanin stability, except for the peonidin derivative in LR. This is consistent with studies indicating that non-aromatic aliphatic acids, lacking a hydrogen bonding network, have a limited ability to form stabilizing complexes with anthocyanins [33]. Differences in aglycone structure and acylation likely account for the cultivar-specific response to organic acids. Consequently, the same copigment may exhibit different affinities for different anthocyanins. The results confirmed that the effect of acids on anthocyanin stability largely depends on the phenolic matrix of a given potato cultivar and cannot be explained solely by pH-dependent stabilization of the flavylium cation. These findings suggest that the hydroxylation and acylation patterns of anthocyanin aglycones influence the response to organic acids [16,31,33,49].

The research conducted and the results obtained can be considered innovative, paving the way for wider use in the food industry of various potato varieties with colored flesh, which contain more phenolic compounds than varieties with traditional yellow, cream, or white flesh. The chemical composition and other characteristics of the potatoes studied indicated that they are particularly well-suited for food processing. However, some of the acids used—particularly lactic and malic acids—have so far been little studied in terms of their interactions with acylated anthocyanins found in red- and purple-fleshed potato varieties.

### 2.4. Effect of Organic Acid Type and Potato Cultivar Used on the Content and Phenolic Acid Profiles of Dehydrated Potato Grits

The control dehydrated potato grits produced from red-fleshed Lily Rose potatoes contained over 2.5 times more phenolic acids than those produced from purple-fleshed Double Fun tubers: 3382 µg·g^−1^ DW and 1317 µg·g^−1^ DW, respectively (Table 5). Chlorogenic acid dominated in all dehydrated potatoes tested and, depending on the type of acid used, constituted from 70 to 71% (LR cultivar) and from 75 to 84% (DF cultivar).

Each of the organic acids used in the production of dehydrated potato grits from DF tubers had a stabilizing effect on phenolic acids. As a result, higher levels of phenolic acids were observed in the dehydrated potatoes produced using organic acids compared to the control sample.

Tartaric acid had a particularly positive effect on the chlorogenic acid content in dehydrated potato grits from DF potatoes, while lactic acid had a significantly negative effect on the chlorogenic acid content in dehydrated potatoes from LR tubers. The obtained results indicate that both potato cultivars and the type of organic acid applied significantly influence the content of phenolic acids in dehydrated potato grits. The most beneficial effect on maintaining a high content of phenolic compounds in the dehydrated potato grits of the Lily Rose cultivar was observed for malic acid, whereas in the case of the Double Fun cultivar, the highest effectiveness was recorded for tartaric acid. The content of coumaric acid in the experimental grits depended largely on the potato cultivar. Grits obtained from LR tubers contained on average 20 times more of this acid than grits from DF tubers. Tartaric acid used in processing DF tubers and malic acid used in processing LR tubers contributed most to the increase in the content of neochlorogenic and cryptochlorogenic acids in the obtained dehydrated potato grits. This indicates a greater susceptibility of these compounds to processing conditions and the applied acidifying agents. The sensitivity of caffeic acid esters, which include the aforementioned chlorogenic acid, has been previously described by Clifford [50].

In summary, the results indicate that the appropriate selection of organic acid can effectively promote the stabilization and preservation of phenolic acids in dehydrated potato grits. The highest effectiveness was demonstrated by malic acid for the Lily Rose cultivar and tartaric acid for the Double Fun cultivar. Breeding research and studies analyzing the processing quality and intended use of new potato cultivars—including those with colored flesh—are still ongoing. The food industry requires specific morphological characteristics of the tubers, as well as specific organoleptic properties and levels of starch, sugars, and non-starch polysaccharides. Therefore, research using appropriate organic acids is necessary to adapt these acids to potato cultivars selected for processing.

## 3. Materials and Methods

### 3.1. Raw Materials

For the study, tubers of two potato cultivars with colored flesh were sampled: Lily Rose (LR), with red flesh, and Double Fun (DF), with purple flesh. The tubers were harvested from the field at full maturity, free from mechanical damage, greening, diseases, and shoots. Samples of 20 kg of tubers of each cultivar were collected. The raw material came from crops grown in the Kuyavian-Pomeranian Voivodeship in Poland during the 2022 growing season and was purchased directly from a producer. The LR cultivar tubers had average dimensions of 60 mm × 80 mm (width × length), while the DF cultivar tubers had average dimensions of 60 mm × 90 mm.

Five analytically pure organic acids were used in the production of grits from this raw material: citric acid (CAS no.: 77-92-9; Sigma-Aldrich, Burlington, MA, USA), lactic acid (CAS no.: 50-21-5; Sigma-Aldrich, Burlington, MA, USA), L-ascorbic acid (CAS no.: 50-81-7; Sigma-Aldrich, Burlington, MA, USA), malic acid (CAS no.: 6915-15-7; Sigma-Aldrich, Burlington, MA, USA), and L-tartaric acid (CAS no.: 87-69-4; Sigma-Aldrich, Burlington, MA, USA).

### 3.2. Experimental Procedure for Manufacturing Dehydrated Potato Grits

Washed, unpeeled potatoes intended for the preparation of experimental dehydrated potato grits were cut into 1.0 cm slices using a mechanical slicer (Robot coupe CL50, Robot Coupe, Vincennes, France). The slices were then blanched in water at 75 °C for 10 min. The hot slices were subsequently cooled in ice water for 2 min. After cooling, the slices were divided into portions and immersed in parallel in either water (control sample) or in 1% solutions of five organic acids (citric acid, lactic acid, L-ascorbic acid, malic acid, and L-tartaric acid) for 5 min. The treated slices were then freeze-dried, ground in a laboratory grinder (Retsch GM 200, Retsch, Haan, Germany) and then passed through a sieve with a mesh size of 1 mm × 1 mm [29].

### 3.3. Basic Analyses of Raw Materials and Ready Product

The starch, reducing sugar, and total protein contents [51] of fresh, unpeeled potato tubers and freeze-dried samples were determined using a standard chemical method. The dry matter content of both the potatoes and experimental grits obtained from them was determined by measuring weight loss after drying to constant mass [51].

### 3.4. Preparation of Extracts for Determination of Anthocyanins, Phenolic Acids and Antioxidant Capacity

Dry samples of unpeeled potatoes from both cultivars, ground using a laboratory electric mill, as well as the experimental grits, constituted the samples in which anthocyanin and phenolic acid contents and profiles were determined. For extract preparation, 1 g of sample was weighed, and 10 mL of 70% methanol was added. The mixture was vortexed for 5 s and extracted in an ultrasonic bath (UM-2, Unitra-Unima, Olsztyn, Poland) for 30 min, followed by shaking for 60 min. This sample was then stored under refrigerated conditions for 20 h, shaken again for 90 min and centrifuged (MPW-351R, Mpw Med. Instruments, Warsaw, Poland) for 10 min at 10,000 rpm and 4 °C. The samples were cleaned using filters (0.45 µm) before application to the column. The resulting supernatant was collected for further analysis [52].

### 3.5. HPLC Analysis of Anthocyanins and Phenolic Acids

The anthocyanin content in potatoes and experimental grits was determined using high-performance liquid chromatography with photodiode array detection (HPLC-PDA) as described by Nemś et al. [13]. Two milliliters of extract samples were filtered into dark glass vials and analyzed by HPLC. A Dionex HPLC system Thermo Fisher Scientific, Germering, Germany) with an Ultimate 3000 diode array detector, an LPG-3400A quaternary pump, an EWPS-3000SI autosampler, and a TCC-3000SD thermostated column chamber controlled by Chromeleon v.6.8 software (Thermo Scientific Dionex, Sunnyvale, CA, USA) was used. Anthocyanins and phenolic acids were determined using a reverse-phase Cadenza Imtakt CD-C18 column (75 mm × 4.6 mm, 5 μm) with a guard column (Imtakt, Kyoto, Japan). The mobile phase consisted of 4.5% formic acid in water and acetonitrile [13]. HPLC analysis was carried out under the following conditions: 0–1 min, isocratic elution with 5% acetonitrile; 1–6 min, linear gradient from 5% to 10% acetonitrile; 6–12 min, linear gradient from 10% to 20% acetonitrile; 26–33 min, linear gradient from 20% to 100% acetonitrile; followed by a return to the initial conditions. The injection volume was 40 μL, and the flow rate was 1 mL/min at a column temperature of 30 °C. Anthocyanins were detected at 520 nm. The HPLC conditions for phenolic acid analysis were analogous to those used for anthocyanin separation, except for the detection wavelength. Phenolic acids were monitored at 320 nm [53]. Anthocyanin content was expressed as mg Cyanidin-3-*O*-glucoside equivalents per 100 g dry weight (DW), whereas phenolic acid content was expressed as μg 5-*O*-caffeoylquinic acid (chlorogenic acid) equivalents per 1 g dry weight (DW).

### 3.6. Total Polyphenol and Flavonoid Contents as Well as Antioxidant Capacity of Raw Material and Dehydrated Potato Grits

The total phenolic content was examined in methanol extracts using the Folin–Ciocalteu colorimetric method as described by Klymenko et al. [52]. Results were expressed as mg gallic acid equivalent (GAE) per 100 g of dry weight (DW) of potatoes or experimental grits. The total flavonoid content was analyzed in methanol extracts, performed using a spectrophotometric method developed by Kim et al. [54], with modifications described by Nemś et al. [13]. Results were expressed as mg catechin equivalents (CE) per 100 g of dry weight (DW) of potatoes or dehydrated potato grits obtained from them. Antioxidant activity was determined in methanolic extracts using the Trolox equivalent antioxidant capacity (TEAC) assay based on ABTS radical cation scavenging according to Re et al. [55], the DPPH radical scavenging assay according to Yen and Chen [56] with modifications described by Klymenko et al. [52], and the ferric reducing antioxidant power (FRAP) assay according to Benzie and Strain [57]. Results were expressed as μmol Trolox equivalents per g dry weight (DW) of potatoes or dehydrated potato grits.

### 3.7. Statistics

In the statistical analysis of the results regarding the chemical composition of potatoes used in the experiment, one-way analysis of variance (ANOVA) and Tukey’s test were used with significance of differences at the level *p* < 0.05. Differences in total polyphenol content, anthocyanin content, and antioxidant capacity (ABTS, DPPH, and FRAP) among dehydrated potato grits obtained from the two cultivars (Lily Rose and Double Fun) after treatment with different organic acids (control, citric, lactic, ascorbic, malic, and tartaric acids) were analyzed using two-way ANOVA. Organic acid treatment and potato cultivar were considered fixed factors, and differences were considered statistically significant at *p* < 0.05. Given the differences in the polyphenolic compound profiles of the Lily Rose and Double Fun cultivars, one-way ANOVA was used to compare the contents of the analyzed polyphenolic compounds, with the level of significance set at *p* < 0.05. Post hoc comparisons were performed using Tukey’s HSD test to identify significant pairwise differences. Statistical analyses were performed using Statistica version 13.1 (StatSoft, Tulsa, OK, USA) [58]. All the measurements were performed in six replicates (two technical repetitions × three laboratory repetitions), and the results are presented as mean values.

## 4. Conclusions

Red-fleshed Lily Rose (LR) and purple-fleshed Double Fun (DF) potatoes responded differently to the organic acid treatments. In general, the resulting dehydrated potato grits contained higher levels of anthocyanins and phenolic acids and exhibited greater antioxidant activity than the untreated controls, although the effects depended on the cultivar and the acid applied. Chlorogenic acid was the predominant phenolic acid, whereas Pelargonidin-3-*p*-coumaroylrutinoside-5-glucoside and Petunidin-3-*p*-coumaroylrutinoside-5-glucoside were the major anthocyanins in LR and DF potatoes, respectively. Malic acid was the most effective in preserving anthocyanins in LR potato grits, while tartaric acid produced the most favorable results in DF products. In contrast, lactic acid adversely affected anthocyanin retention in LR potato grits. These cultivar-dependent responses indicate that the effectiveness of organic acid treatments is associated with the anthocyanin profile of the raw material, including the structure and substitution pattern of the predominant pigments. However, the results do not provide evidence that organic acids acted directly as copigments. Based on the information available in the literature, the effect of organic acids can be attributed to changes in physicochemical conditions during the processing of raw materials, such as pH, whose stabilizing effect on anthocyanins has been confirmed by many authors. Overall, these findings demonstrate the importance of selecting organic acid treatments according to potato cultivar and anthocyanin profile to improve pigment retention and preserve the antioxidant potential of dehydrated potato grits. Access to cultivars with colored flesh, differing in aglycones, and possessing characteristics suitable for industrial use is limited and requires further research, including cultivation and storage studies.

## Figures and Tables

**Table 1:** Raw material characteristics of potatoes of two potato cultivars with colored flesh used in the experiment.

Feature	Potato Cultivar	
	Lily Rose (LR)	Double Fun (DF)
Dry matter [g·100 g^−1^ FW]	19.90 a ± 0.07	20.14 a ± 0.19
Starch [g·100 g^−1^ FW]	15.10 b ± 0.13	15.39 a ± 0.11
Total proteins [g·100 g^−1^ FW]	2.45 a ± 0.06	2.62 a ± 0.07
Reducing sugars [g·100 g^−1^ FW]	0.12 a ± 0.10	0.09 b ± 0.08
Total polyphenols [mg GAE·100 g^−1^ DW]	374.00 b ± 1.90	437.90 a ± 2.10
Total flavonoids [mg CE·100 g^−1^ DW]	303.50 a ± 2.73	225.70 b ± 2.04
DPPH [µmol Trolox·g^−1^ DW]	22.55 b ± 0.44	25.55 a ± 0.72
ABTS [µmol Trolox·g^−1^ DW]	83.18 a ± 3.47	88.38 a ± 2.99
FRAP [µmol Trolox·g^−1^ DW]	8.41 b ± 0.25	14.15 a ± 0.36

Different letters within each row (a, b) indicate significant differences for each compound at *p* < 0.05; values are expressed as mean ± SD (standard deviation); *n* = 6; FW—fresh weight; DW—dry weight.

**Table 2:** Anthocyanin and phenolic acid profiles of unpeeled potatoes of two cultivars.

Compounds	Potato Cultivar	
	Lily Rose (LR)	Double Fun (DF)
**Anth** [mg Cy 3-glc 100 g^−1^ DW]		
Pg 3-rut-5-glc	2.06 b ± 0.5.0	n.d.
Pn der	0.94 c ± 0.05	n.d.
Pn 3-(coum)rut-5-glc	0.54 c ± 0.01	n.d.
Pg 3-(coum)rut-5-glc	46.20 a ± 1.60	n.d.
Pg 3-(fer)rut-5-glc	3.99 b ± 0.08	n.d.
Pt 3-(caf)rut-5-glc	n.d.	5.02 bc ± 0.81
Pt 3-(coum)rut-5-glc	n.d.	188.70 a ± 20.74
Pt der	n.d.	0.16 c ± 0.05
Mv 3(coum)rut-5-glc	n.d.	30.28 b ± 3.69
Other, ni Anth	7.60	7.54
***Total Anth***	**61.33**	**231.70**
**PhA** [mg·100 g^−1^ DW]		
(3-CQA)	22.16 BC ± 0.46	16.60 B ± 1.98
(5-CQA)	179.70 A ± 7.50	146.90 A ± 14.95
(4-CQA)	32.67 B ± 4.48	15.12 B ± 0.12
*p*-Coum	13.82 C ± 0.655	2.14 C ± 0.32
Other, ni PhA	7.45	7.64
***Total PhA***	**255.80**	**188.40**

Different lowercase letters (a–c) for the Lily Rose cultivar and different uppercase letters (A–C) for the Double Fun cultivar indicate significant differences (*p* < 0.05); ± SD (standard deviation); *n* = 6; DW—dry weight; n.d.—not detected. Abbreviations: Anth: Anthocyanins; Cy 3-glc: Cyanidin-3-*O*-glucoside; Pg 3-rut-5-glc: Pelargonidin-3-rutinoside-5-glucoside; Pn der: peonidin derivative; Pn 3-(coum)rut-5-glc: Peonidin-3-*p*-coumaroylrutinoside-5-glucoside; Pg 3-(coum)rut-5-glc: Pelargonidin-3-*p*-coumaroylrutinoside-5-glucoside; Pg 3-(fer)rut-5-glc: Pelargonidin-3-feruloyl-rutinoside-5-glucoside; Pt 3-(caf)rut-5-glc: Petunidin-3-caffeoylrutinoside-5-glucoside; Pt 3-(coum)rut-5-glc: Petunidin-3-*p*-coumaroylrutinoside-5-glucoside; Pt der: Petunidin derivative; Mv 3(coum)rut-5-glc: Malvidin-3-*p*-coumaroylrutinoside-5-glucoside; Other, ni Anth: Other, not identified anthocyanins; PhA: Phenolic acids; (3-CQA): Neochlorogenic acid; (5-CQA): Chlorogenic acid; (4-CQA): Cryptochlorogenic acid; *p*-Coum*: p*-Coumaric acid; Other, ni PhA: Other, not identified phenolic acids.

**Table 3:** Results of ANOVA and Tukey’s HSD tests for the effects of organic acid type and potato cultivar on the content of polyphenols and flavonoids and antioxidant activity of the obtained potato products.

Factor	Total Polyphenols	Total Flavonoids	DPPH	ABTS	FRAP
	[mg GAE·100 g^−1^ DW]	[mg CE·100 g^−1^ DW]	[µmol Trolox·g^−1^ DW]		
**ANOVA Test**					
Potato cultivar	***	***	***	***	***
Acid type	***	***	***	***	***
**HSD test**					
Type of acid					
Control	335.1 c ± 73.1	244.9 b ± 56.6	22.36 d ± 0.47	78.34 d ± 7.77	15.20 c ± 3.17
Citric acid	496.5 b ± 94.4	331.2 a ± 8.9	29.34 b ± 6.43	91.92 c ± 9.97	14.33 de ± 5.76
Lactic acid	512.3 b ± 1.18	267.8 b ± 39.1	26.68 c ± 10.24	93.69 c ± 21.06	13.93 e ± 9.45
Ascorbic acid	585.5 a ± 110.0	313.1 a ± 20.7	31.35 ab ± 5.68	108.54 a ± 13.66	23.12 ab ± 8.94
Malic acid	523.4 b ± 120.1	330.3 a ± 59.1	33.20 a ± 4.74	102.73 b ± 4.28	18.87 b ± 4.19
Tartaric acid	565.8 a ± 177	334.7 a ± 95.6	29.56 b ± 7.19	101.65 b ± 22.59	24.41 a ± 10.68
Potato cultivar					
Lily Rose	442.6 b ± 35.8	290.1 b ± 32.6	23.47 b ± 3.40	86.73 b ± 9.43	11.76 b ± 3.65
Double Fun	630.9 a ± 128.2	340.2 a ± 67.1	34.03 a ± 5.93	105.57 a ± 17.46	24.86 a ± 6.83

*** significant at *p* < 0.001; *n* = 6; a–e—values followed by the same letter within the same column were not significantly different, according to Tukey’s least significant differences test; ±SD—standard deviation; DW—dry weight.

**Table 4:** Anthocyanin contents and profiles [mg Cyanidin-3-o-glucoside ·100 g^−1^ DW] of dehydrated potato grits made from Lily Rose and Double Fun tubers, depending on the organic acid used.

Potato Cultivar	Anthocyanins	Type of Acid					
		Control	Citric	Lactic	Ascorbic	Malic	Tartaric
Lily Rose	Pg 3-rut-5-glc	1.78 b ± 0.46	2.78 b ± 0.16	0.42 c ± 0.08	2.37 b ± 0.27	4.49 a ± 0.21	2.76 b ± 0.34
	Pn der	0.78 b ± 0.17	0.59 bc ± 0.09	1.57 a ± 0.03	0.73 b ± 0.11	1.37 a ± 0.00	0.47 bc ± 0.27
	Pn 3-(coum)rut-5-glc	0.67 b ± 0.17	0.91 ab ± 0.07	0.59 b ± 0.11	0.90 ab ± 0.20	1.79 a ± 0.15	1.51 ab ± 0.52
	Pg 3-(coum)rut-5-glc	56.09 b ± 2.37	57.95 ab ± 0.25	40.99 c ± 0.09	56.68 ab ± 2.98	65.90 a ± 1.69	54.04 b ± 4.12
	Pg 3-(fer)rut-5-glc	3.17 ab ± 1.61	2.57 ab ± 0.02	0.62 c ± 0.16	1.93 ab ± 0.24	3.64 a ± 0.18	2.46 ab ± 0.29
	Other, ni Anth	8.79	9.69	6.50	9.19	12.71	8.42
	*Total Anth*	71.28 b	74.49 b	50.69 c	71.80 b	89.90 a	69.66 b
Double Fun	Pt 3-(caf)rut-5-glc	2.14 D ± 0.64	10.27 C ± 0.03	11.65 C ± 0.00	15.31 B ± 0.51	14.87 B ± 0.03	26.02 A ± 0.74
	Pt 3-(coum)rut-5-glc	115.10 D ± 11.88	245.60 C ± 1.77	263.00 C ± 1.08	318.60 AB ± 4.52	308.50 B ± 3.31	346.10 A ± 10.99
	Pt der	1.25 B ± 0.17	0.31 C ± 0.01	0.18 C ± 0.00	1.17 B ± 0.05	1.33 B ± 0.06	2.16 A ± 0.08
	Mv 3(coum)rut-5-glc	17.23 C ± 2.32	35.01 B ± 0.13	35.03 B ± 0.09	38.95 AB ± 0.53	40.01 A ± 0.21	41.91 A ± 1.17
	Other, ni Anth	5.18	8.11	8.04	7.97	7.79	12.11
	*Total Anth*	140.90 D	299.30 C	317.90 C	382.00 B	372.50 B	428.30 A

Different lowercase letters (a–c) for the Lily Rose cultivar and different uppercase letters (A–D) for the Double Fun cultivar indicate significant differences (*p* < 0.05); values are presented as mean ± SD (standard deviation); *n* = 6; DW—dry weight. Abbreviations: Anth: Anthocyanins; Pg 3-rut-5-glc: Pelargonidin-3-rutinoside-5-glucoside; Pn der: Peonidin derivative; Pn 3-(coum)rut-5-glc: Peonidin-3-*p*-coumaroylrutinoside-5-glucoside; Pg 3-(coum)rut-5-glc: Pelargonidin-3-*p*-coumaroylrutinoside-5-glucoside; Pg 3-(fer)rut-5-glc: Pelargonidin-3-feruloyl-rutinoside-5-glucoside; Other, ni Anth: Other, not identified anthocyanins; Pt 3-(caf)rut-5-glc: Petunidin-3-caffeoylrutinoside-5-glucoside; Pt 3-(coum)rut-5-glc: Petunidin-3-*p*-coumaroylrutinoside-5-glucoside; Pt der: Petunidin derivative; Mv 3(coum)rut-5-glc: Malvidin-3-*p*-coumaroylrutinoside-5-glucoside; Other, ni Anth: Other, not identified anthocyanins.

**Table 5:** Phenolic acid contents and profiles [µg·g^−1^ DW] of dehydrated potato grits made from Lily Rose (LR) and Double Fun (DF) tubers, depending on the organic acid used.

Potato Cultivar	Phenolic Acid	Organic Acid Used					
		Control	Citric	Lactic	Ascorbic	Malic	Tartaric
Lily Rose (LR)	(3-CQA)	263.6 b ± 19.80	276.6 ab ± 17.71	154.7 c ± 7.79	275.2 ab ± 18.78	340.5 a ± 7.24	290.8 ab ± 21.25
	(5-CQA)	2496 a ± 198.08	2531 a ± 56.81	1786 b ± 94.73	2462 a ± 225.84	2987 a ± 80.44	2426 a ± 162.59
	(4-CQA)	337.8 a ± 7.00	332.5 ab ± 13.51	222.8 b ± 10.69	323.4 ab ± 59.84	415.8 a ± 28.89	350.9 a ± 13.14
	*p*-Coum	379.8 a ± 28.60	324.4 a ± 57.71	278.7 a ± 10.80	369.4 a ± 15.14	387.6 a ± 16.60	302.5 a ± 28.2
	Other, ni PhA	95.2	79.5	80.8	80.0	79.1	77.8
	*Total PhA*	3382 b	3544 b	2523 c	3510 b	4210 a	3448 b
Double Fun (DF)	(3-CQA)	86.02 D ± 8.74	302.7 C ± 3.35	304.4 C ± 1.10	330.1 BC ± 8.25	351.8 B ± 4.75	441.2 A ± 13.67
	(5-CQA)	1061 C ± 47.61	2600 AB ± 35.29	2485 B ± 61.76	2828 AB ± 131.3	2930 AB ± 107.9	3026 A ± 181.4
	(4-CQA)	94.56 C ± 39.38	357.8 AB ± 31.27	140.3 BC ± 198.42	394.6 AB ± 41.62	388.0 AB ± 40.33	486.5 A ± 18.63
	*p*-Coum	17.05 A ± 24.11	20.02 A ± 0.58	18.62 A ± 0.59	14.97 A ± 0.19	13.98 A ± 0.59	15.08 A ± 3.50
	Other, ni PhA	58.4	40.5	18.7	50.3	50.2	47.2
	*Total PhA*	1317 E	3321 C	2967 D	3618 B	3734 B	4016 A

Different lowercase letters (a–c) for the Lily Rose cultivar and uppercase letters (A–E) for the Double Fun cultivar within the same column indicate significant differences (*p* < 0.05); values are presented as mean ± SD (standard deviation); *n* = 6; DW—dry weight. PhA: Phenolic acids; (3-CQA): Neochlorogenic acid; (5-CQA): Chlorogenic acid; (4-CQA): Cryptochlorogenic acid; *p*-Coum: *p*-Coumaric acid; Other, ni PhA: Other, not identified phenolic acids; Total PhA: Total phenolic acids.

## Data Availability

The datasets generated for this study are available from the authors of the manuscript.
